# Four-channel graphene optical receiver

**DOI:** 10.1515/nanoph-2024-0274

**Published:** 2024-07-31

**Authors:** Laiwen Yu, Yurui Li, Hengtai Xiang, Yuanrong Li, Hengzhen Cao, Zhongyang Ji, Liu Liu, Xi Xiao, Jianbo Yin, Jingshu Guo, Daoxin Dai

**Affiliations:** State Key Laboratory of Extreme Photonics and Instrumentation, College of Optical Science and Engineering, International Research Center for Advanced Photonics, 12377 Zhejiang University, Zijingang Campus, Hangzhou 310058, China; State Key Laboratory of Advanced Optical Communications System and Networks, School of Electronics, Peking University, Beijing 100871, P.R. China; National Information Optoelectronics Innovation Center, China Information and Communication Technologies Group Corporation (CICT), Wuhan 430074, China; Jiaxing Key Laboratory of Photonic Sensing & Intelligent Imaging, Intelligent Optics & Photonics Research Center, 12377 Jiaxing Research Institute, Zhejiang University, Jiaxing 314000, China; Academy for Advanced Interdisciplinary Studies, Peking University, Beijing 100871, China; Beijing Graphene Institute, Beijing 100095, P.R. China

**Keywords:** silicon photonics, graphene photodetectors, photo-thermoelectric effect, optical receiver, wavelength division multiplexing

## Abstract

Silicon photonics with the advantages of low power consumption and low fabrication cost is a crucial technology for facilitating high-capacity optical communications and interconnects. The graphene photodetectors (GPDs) featuring broadband operation, high speed, and low integration cost can be good additions to the SiGe photodetectors, supporting high-speed photodetection in wavelength bands beyond 1.6 μm on silicon. Here we realize a silicon-integrated four-channel wavelength division multiplexing (WDM) optical receiver based on a micro-ring resonator (MRR) array and four p-n homojunction GPDs. These photo-thermoelectric (PTE) GPDs exhibit zero-bias responsivities of ∼1.1 V W^−1^ and set-up limited 3 dB-bandwidth >67 GHz. The GPDs show good consistence benefiting from the compact active region array (0.006 mm^2^) covered by a single mechanically exfoliated hBN/graphene/hBN stack. Moreover, the WDM graphene optical receiver realized 4 × 16 Gbps non-return-to-zero optical signal transmission. To the best of our knowledge, it is the first GPD-array-based optical receiver using high-quality mechanically exfoliated graphene and edge graphene-metal contacts with low resistances. Apparently, our design is also compatible with CVD-grown graphene. This work sheds light on the large-scale integration of GPDs with high consistency and uniformity, enabling the application of high-quality mechanically exfoliated graphene, and promoting the development of the graphene photonic integrated circuits.

## 1 Introduction

The ever-growing demand of global data traffic is driving the development of next-generation optical communication and interconnection technologies, standards and modules such as transmitters and receivers [1], [2]. Silicon photonics has attracted great interests due to its high integration density and low cost benefiting from the complementary metal-oxide-semiconductor (CMOS) fabrication process [2]. With the help of wavelength division multiplexing (WDM) technology, the silicon photonic transmitters and receivers have been widely used in optical interconnect in data centers and optical communications [2], [3]. Currently, the silicon photonic optical receivers mainly working at O-band or C + L-bands, and typically use the SiGe photodetectors (PDs) [4]. Extending the optical communication bands is a direct and effective solution for capacity improvement [5], e.g., the U-band (1.625 μm–1.675 μm) communication has received extensive attention currently [6]. However, Ge usually has a cut-off wavelength of ∼1.6 μm for efficient optical absorption [4], when no special process is used. Meanwhile, the integration of III–V PDs on Si needs bonding fabrication technology [7] or special integration technology [8] in cost of larger cost and lower yield. Therefore, the high-speed and CMOS-compatible silicon integrated PDs operating in a wide band (e.g., from near-infrared to mid-infrared) are in urgent demand. Fortunately, graphene can provide a promising solution for its ultrafast carrier dynamics [9], high carrier mobility [10], and broadband photoresponse [11]. Moreover, as a two-dimensional material, graphene can be integrated onto photonics platforms without lattice mismatch, and can even be compatible with CMOS back-end line processing [12].

Recently, large-bandwidth graphene photodetectors (GPDs) have been demonstrated using photoconductor or phototransistor structures based on PV effect [13], bolometric (BOL) effect [14]–[16], and photoconductive (PC) effect [14], the G-Si heterostructure based on IPE effect [17], and the p-n homojunction structures based on the photothermoelectric (PTE) effect [18–24]. The G-Si heterostructure PDs typically exhibit limited quantum efficiency. Photoconductors/phototransistors tend to suffer from substantial dark current (∼mA scale under bias). In contrast, zero-biased p-n homojunction GPDs based on the PTE effect (PTE GPDs) have emerged as the preferred approach for high-speed GPDs development due to their superior signal-to-noise ratios [11]. However, the sensitivity and linearity of the PTE GPDs still have a significant gap compared to Si–Ge PDs. Better quality graphene [25] and graphene-metal contacts [26] are urgently needed to improve the responsivity and reduce the thermal noise of the PTE GPDs.

The integration methods of graphene on photonic platforms have two ways. The CVD-grown graphene is usually used by wet-transfer [27] or semi-dry transfer [28] methods, supporting large-scale (e.g., wafer-scale [29]) photonic integration. In terms of the PTE PDs using CVD-grown graphene, the state-of-the-art works have demonstrated 105 Gbps of single-device direct detection speed [24], the bandwidth of >67 GHz [21], and the responsivity of 10 V W^−1^ [20]. Alternatively, mechanical exfoliation can be used to transfer graphene from high-quality bulk graphite using adhesive tapes without water or solvent involved [27]. This method results in graphene with superior material quality compared to CVD-grown graphene, specifically exhibiting higher mobility and fewer impurities. Moreover, the mechanical exfoliated graphene can usually be encapsulated by hBN/graphene/hBN stack structure [18] to realize better stability and mobility of graphene, and the edge graphene-metal contact can be realized with much lower resistance. In a representative work, a record responsivity of 90 V W^−1^ was reported [22] using the stack of hBN/graphene/hBN integrated on MRRs. While CVD-grown graphene has been applied to construct several active photonic integrated circuits (PICs) [16], [30], the photonic devices based on mechanical exfoliated graphene have only been used in single photonic device to the best of our knowledge due to the limited transfer area.

In this work, we propose and demonstrate an integrated four-channel WDM optical receiver based on MRR wavelength demultiplexing filters and PTE GPDs with mechanically exfoliated monolayer graphene. A large hBN/single layer graphene (SLG)/hBN heterostructure is transferred by the mechanical exfoliation method onto a specially designed active region array with total footprint of only 100 × 60 μm^2^. The four PTE PDs are based on thin-silicon slot-waveguide as our previous work introduced [23]. When operating at 1,550.25/1,548.7/1,546.4/1,544.68 nm, the present GPDs achieve responsivities of ∼1.1 V W^−1^ and flat frequency response up to 67 GHz (set-up limited). The linear dynamic ranges are measured from 0.01 mW to 0.4 mW under zero current bias. To the best of our knowledge, this is the first work using mechanically exfoliate graphene to realize arrayed GPDs, based on which a WDM optical receiver supporting 4 × 16 Gbps NRZ transmission is demonstrated. In this work, the wavelength band of ∼1.55 μm is chosen for convenience of device test. This work can be easily extended to longer wavelengths (e.g., U-band, 2-μm-band [23] and beyond).

## 2 Structure and design

**Figure 1:**
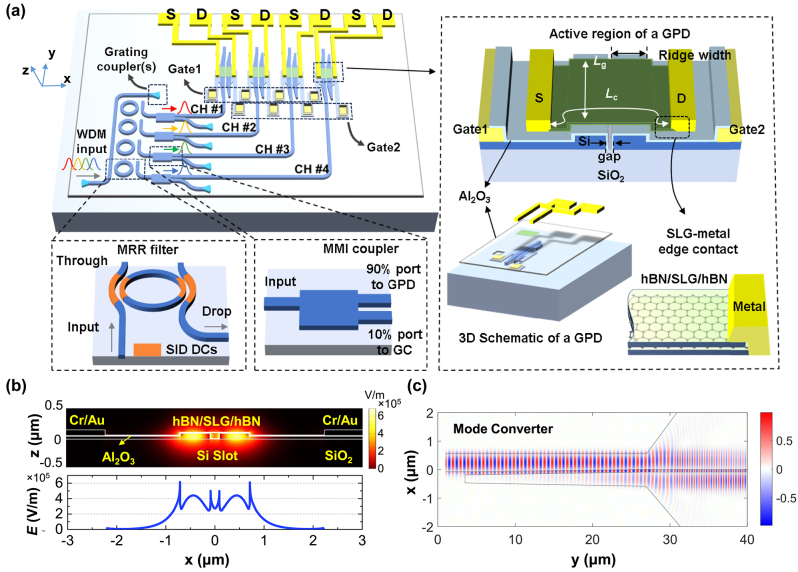
Design of the present four-channel WDM optical receiver based on GPDs. (a) Schematic configuration of the photonic integrated circuit with grating couplers, MRRs, MMIs and the GPDs. Inset: Structures of a MRR, a MMI, the structure of a GPD, and the SLG-metal edge contact. *L*_c_, channel length; *L*_g_, channel width; SLG, single layer graphene; CH, channel; SID, semi-inverse design; DC, directional coupler. (b) Transversal electric field distributions $\left(\left|E_{T}\right|=\sqrt{{\left|E_{x}\right|}^{2}+{\left|E_{y}\right|}^{2}}\right)$ of the fundamental quasi-TE mode for the optimized slot ridge waveguide and graphene sheet, the normalized mode power is 1 mW. (c) Normalized simulated light propagation in the mode converter when operating at 1.55 μm.

Figure 1 illustrates the working principle of the proposed graphene-based optical receiver, which is composed of a 4-channel wavelength demultiplexing filter and four PTE GPDs. All the receiver is based on the 100-nm-thick silicon waveguide platform introduced in our previous work [23]. Light can be coupled into the fundamental TE mode of the waveguide through the fiber-to-chip grating coupler, and then demultiplexed by the four MRRs to channels #1–#4. The semi-inverse-designed (SID) method [31] was used to design the broadband, low loss, fabrication-friendly directional couplers, which are used to construct the high-performance MRRs with good fabrication tolerances. In a free spectral range (FSR), the MRRs of channels #1–#4 correspond to the drop-port central wavelengths of 1,550.25 nm, 1,548.7 nm, 1,546.4 nm and 1,544.68 nm, respectively.

For each channel #*x*, the optical signal is transmitted through a MRR and a multimode interferometer (MMI) with a power splitting ratio of 90 %/10 %. The 10 % port is connected to an output grating coupler for optical coupling alignment. The 90 % power optical signal is then input to the corresponding GPD #*x*. The theoretical losses of the grating coupler, MRR, and MMI at the center wavelength are 7, ∼0.5, and ∼0.5 dB. In each GPD, the injected optical TE_0_ mode is converted to the fundamental quasi-TE mode of the slot waveguide where the active region locates by a strip-to-slot-waveguide mode converter., As Figure 1(a) shows, *L*_c_ and *L*_g_ are the channel length and width of the GPD, respectively. To ensure all the four active regions can be covered by the mechanically exfoliated hBN/SLG/hBN stack, we designed a compact active region array with a footprint of <100 × 60 μm^2^. The spacing between the adjacent GPDs are ∼4.6 μm. All the GPDs share the same structure which is like our previous work [23]. In the thin-Si slot waveguide of the active region, two metal (Al/Au) pads respectively connect the left and right silicon regions, acting as the gate electrodes. The mechanically exfoliated hBN/SLG/hBN stack is positioned on top of the 10-nm-thick Al_2_O_3_ dielectric insulator layer that encases the Si waveguide. As the most popular encapsulation material for graphene, hBN can enhance the mobility of graphene [18]. In this way, the doping level of the graphene-sheet can be modulated separately by the two gate electrodes. The one-dimensional edge contacts between the source and drain (Cr/Au) electrodes and graphene are formed.

The active region waveguide is optimized for efficient optical absorption in graphene, resulting in parameters of ridge width = 0.6 μm, gap = 0.15 μm. Figure 1(b) depicts the fundamental quasi-TE mode field simulated by COMSOL with the effective refractive index of 1.902–0.0043*i* at 1.55 μm, corresponding to a mode absorption coefficient of about ∼0.15 dB μm^−1^ [23], [32]. The loss of the bounded mode can be all attributed to the graphene absorption. Figure 1(c) shows the simulated light propagation field indicating the strip-to-slot mode conversion process. The strip-to-slot-waveguide mode converter is designed by 3D-FDTD simulation (Lumerical Inc.) with a coupling loss of ∼0.7 dB at 1.55 μm. The design details of the MRRs, MMI, and mode converter are introduced in Supplementary Note 1.

**Figure 2:**
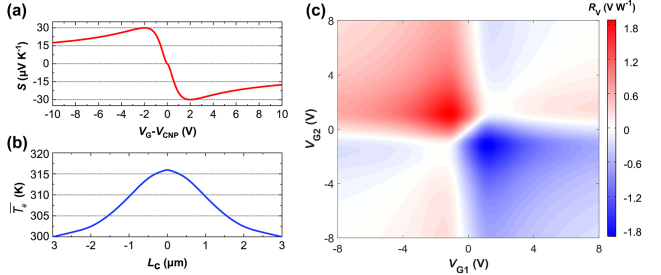
Simulation results of the PTE process. (a) Calculated seebeck coefficient as a function of gate voltage. (b) Calculated lengthwise-average electron temperature profile along the graphene channel between the source and drain contacts (*P*_in_ = 0.1 mW). (c) Six-fold photovoltage responsivity map of the designed GPD at 1.55 μm with *L*_c_ = 4.5 μm and *L*_g_ = 30 μm, *P*_in_ = 0.1 mW. *P*_in_: input optical power to the GPD.

The simulation of PTE processing has been discussed in our previous work [14], [23]. The channel length is set to *L*_c_ = 4.5 μm, taking account the PTE responsivity and active region footprint control, while the channel width is set to *L*_g_ = 30 μm based on the two-dimensional material (2DM) stack size. The resistance with varied gate voltage of a reference FET (field effect transistor) structure was fabricated along with similar technology and measured to get the resistance-gate-voltage relation, from which graphene parameters are extracted: the graphene mobility *μ* = ∼5.06 × 10^4^ cm^2^ V^−1^ s^−1^, the minimum conductivity *σ*_min_ = ∼2.6 mS, and cooling length *ζ* = 1 μm (see Supplementary Note 2 for more details). The Seebeck coefficient is given by $S=-\frac{\pi^{2}k_{\text{B}}^{2}T}{3e}\frac{1}{\sigma }\frac{\text{d}\sigma }{\text{d}\mu_{c}}$ , where *e* is the electron charge, *k*_B_ denotes the Boltzmann constant, and *μ*_c_ is the graphene chemical potential. Figure 2(b) shows the calculated Seebeck coefficient as a function of the gate voltage with room temperature *T* = 300 K. The electron temperature distribution *T*_e_(*l*, *y*) was obtained by solving the heat diffusion partial differential equation [14], [23], where *l* is the lateral position along the graphene sheet.

Figure 2(b) depicts the lengthwise-average electron temperature ${\bar{T}}_{e}\left(l\right)$ given by ${\bar{T}}_{e}\left(l\right)=\frac{\int_{0}^{L}T_{e}\left(l,y\right)\text{d}y}{L_{g}}$ with *V*_G1_−*V*_CNP_ = −1 V and *V*_G2_−*V*_CNP_ = 1 V (or vice versa), and the highest temperature is ∼317 °C when the input optical power is *P*_in_ = 0.1 mW at 1.55 μm. In this case, the input optical power of the slot waveguide active region is 0.087 mW given that the mode converter loss is 0.7 dB. The PTE photovoltage *V*_PTE_ was then calculated by $V_{\text{PTE}}=\int S\left(l\right)\frac{\int \text{d}{\bar{T}}_{e}\left(l\right)}{\text{d}l}\text{d}l$ . In this way, the photoresponse under different gate voltages can be simulated. Figure 2(c) shows the gate-voltage-dependent photovoltage responsivity (*R*_v_ = *V*_PTE_/*P*_in_) map under zero bias when *P*_in_ = 0.1 mW at 1.55 μm. The result illustrates a six-fold pattern originating from the typical PTE photoresponse [33], [34]. When *V*_G1_−*V*_CNP_ = −1 V and *V*_G2_−*V*_CNP_ = 1 V or vice versa), the responsivity (*R*_v_ = 1.83 V W^−1^) is maximized.

## 3 Experimental results and analyses

The silicon waveguides were fabricated on a SOI wafer with a 100-nm-thick top-silicon layer and 3-μm-thick buried-oxide layer using the processes of electron-beam lithography (EBL) and inductively coupled plasma (ICP) etching. The thin silicon slot ridge waveguides can enhance the evanescent field vertically and thus enhance the light-graphene interaction. Then, the gate electrodes (Al/Au) are fabricated by electron-beam evaporation (EBE) and lift-off processes, followed by the deposition of a ∼10-nm-thick Al_2_O_3_ insulator layer. The hBN/SLG/hBN stack was transferred onto the active region array by van der Waals assembly technique. The graphene-metal (Cr/Au) edge contacts were fabricated by EBL and EBE (more details are given in Section 5 and Supplementary Note 3).

**Figure 3:**
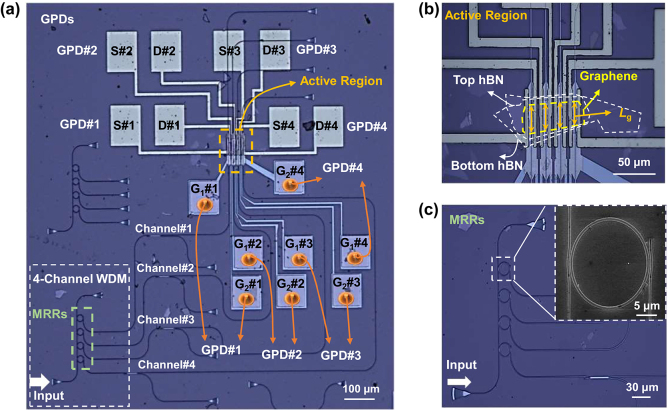
The fabricated 4-channel WDM optical receiver based on PTE GPDs. (a) Microscope picture of the photonic integrated circuit. *S*#*n*, *D*#*n* are respectively the source, drain electrodes of GPD #*n* (*n* = 1, 2, 3, 4). Gates are contacted through metal contacts (G_1_#1–#4 and G_2_#1–#4). Scale bar, 100 μm. (b) Microscope picture of the GPDs with *L*_c_ = 4.5 μm and *L*_g_ = ∼30, ∼30, ∼28, ∼27 μm (GPDs #1–#4). Scale bar, 50 μm. (c) Microscope picture of the MRR array. Scale bar, 30 μm. Insert figure, SEM picture of an add-drop MRR. Scale bar, 5 μm.

Figure 3(a) presents the optical microscope picture of the as-fabricated 4-channel WDM optical receiver fabricated based on MRR array and compact integrated PTE GPDs. Figure 3(b) shows the optical microscope picture and the scanning electron microscope (SEM) picture of the fabricated GPD array. For GPDs #1–#4, the channel length *L*_c_ is 4.5 μm, and the channel width *L*_g_ (i.e., the optical absorption length in active region) is ∼30, ∼30, ∼28, ∼27 μm for the GPD #1, #2, #3, #4. Figure 3(c) shows the fabricated MRRs for wavelength demultiplexing as well as a SEM picture of an add-drop MRR.

**Figure 4:**
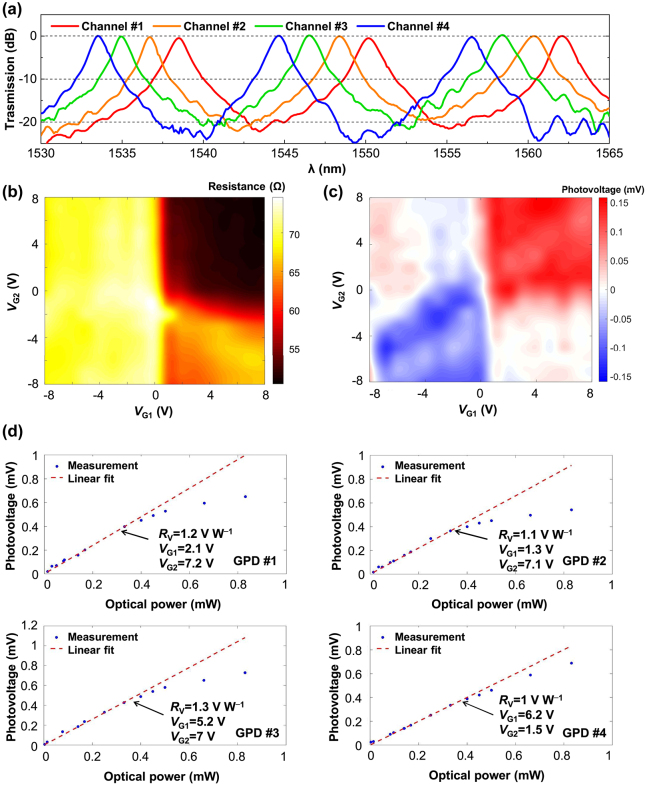
Static experimental results of the MRRs and the GPDs. (a) Measured transmission spectrum of the fabricated MRRs. (b), (c) Measured results of GPD #1 (*L*_c_ = 4.5 μm and *L*_g_ = ∼30 μm) including the resistance map (b) of as a function of the gate voltages *V*_G1_ and *V*_G2_ and the photovoltage map (c) at 1,550.25 nm with *P*_in_ = ∼0.1 mW under zero-current bias. (d) Photovoltage *V*_PTE_ as a function of input optical power *P*_in_ under zero-current bias with the gate voltages *V*_G1_ and *V*_G2_. Dots and dashed lines are respectively the measured results and fitting results governed by *R*_V_ = *V*_PTE_/*P*_in_.

Figure 4(a) shows the measured transmission spectra for the fabricated MRRs. The excess loss (ELs) at the center wavelengths for the four channels are <1 dB with ∼1.5-nm channel spacing. In an FSR, all four channels have a 1-dB bandwidth of ∼0.5 nm and 3-dB bandwidth of ∼0.87 nm. The better performance of the MRRs contributes to the stability of the work of this system. The static performance of the as-fabricated GPDs was measured with three source meters [23]. Figure 4(b) and (c) present the measurement results of GPD #1 with *L*_c_ = 4.5 μm and *L*_g_ = ∼30 μm. The resistance of the graphene channel under different gate voltages was measured under near-zero bias voltage of 1 mV, showing that the charge neutrality point voltage *V*_CNP_ is about −1 V, as shown in Figure 4(b). The optical power input to a GPD, denoted by *P*_in_, is determined by subtracting the passive device losses (including the MRR and MMI) from the output power of the fiber. The experimentally measured losses of the grating couplers, MMR, and MMI are 12, <1, and ∼1 dB, respectively. Figure 4(c) gives the measured *V*_PTE_ under zero current at the wavelength of 1,550.25 nm with *P*_in_ = ∼0.1 mW as a function of the gate voltages (*V*_G1_, *V*_G2_). Figure 4(d) shows the measured relation of *V*_PTE_ ∼ *P*_in_ under zero current for the four GPDs. By fitting the relation of *V*_PTE_ ∼ *P*_in_, the GPD #1 has a responsivity *R*_V_ of ∼1.2 V W^−1^ when *V*_G1_ = 2.1 V, and *V*_G2_ = 7.2 V. As Figure 4(c) shows, the four GPDs have similar responsivities: GPD #2: *R*_V_ = ∼1.1 V W^−1^ when *V*_G1_ = 1.3 V, and *V*_G2_ = 7.1 V; GPD #3: *R*_V_ = ∼1.3 V W^−1^ when *V*_G1_ = 5.2 V, and *V*_G2_ = 7 V; GPD #4: *R*_V_ = ∼1 V W^−1^ when *V*_G1_ = 6.2 V, and *V*_G2_ = 1.5 V. The measured linear dynamic ranges of four GPDs are similar, i.e., 0.01–0.4 mW. When *P*_in_ increases to beyond 0.4 mW, the linearities of the GPDs degrade due to the *T*_e_ dependence of the electronic heat capacity [22], [35]. More details of the static performances of GPDs #2–#4 are given in Supplementary Note 4. The non-ideal distributions in the resistance and photoresponse maps of the GPDs can be attributed to the imperfect gate electrodes. The ohmic contacts between Al and Si is not ideal, coupled with the relatively high resistance of lightly doped thin silicon, may cause discrepancy between the electric potential difference in the silicon-insulator-graphene structure and *V*_G1_ (or *V*_G2_). Moreover, the presence of non-ideal conformal contacts between the 2DM stack and the ridge waveguide platform may also contribute to the imperfect gating control. The variations of *V*_CNP_ and the optimal operation gate voltages of the four GPDs may also be caused by the variations of the as-fabricated gate electrodes. More details of the static performances of GPDs #2–#4 are given in Supplementary Note 4.

**Figure 5:**
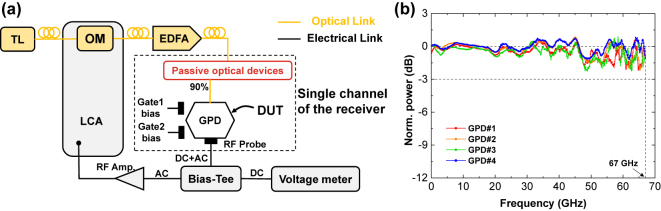
High frequency response of the GPDs. (a) Measurement setup. (b) Normalized high frequency response of the four GPDs with zero-current bias. LCA: light-wave component analyzer, OM: optical modulator, RF Amp.: radio frequency amplifier, EDFA: erbium-doped fiber amplifiers, DC: direct current, AC: alternating current. Passive optical devices include the grating coupler, MRR, and MMI.

As shown in Figure 5(a), the frequency responses of the GPDs were measured by using a commercial light-wave component analyzer (LCA). The high frequency response of the GPD under zero-current bias was obtained from the measured *S*_21_ calibrated by the responses of a RF amplifier and a RF probe. The modulated optical signal was generated by the LCA and was amplified with an erbium-doped fiber amplifier (EDFA). For each WDM channel, the signal was then injected to the GPD through the passive optical devices. Here a bias-Tee was used to apply the zero-current bias and collected the RF response simultaneously. Since the corresponding passive MRR is involved in the reference calibration link with help of the broadband MMI power splitter and the grating couplers, the influence of the narrow-band MRR can also be calibrated. Under an open circuit test mode (zero-current bias), the measured high frequency response (normalized *S*_21_) of the all four GPDs are shown in Figure 5(b) with good consistence. The gate voltages were set to the optimized values according to the static measurements. When the gate voltages change around the optimized values, the normalized S_21_ spectrum of each GPD changes in amplitude with coincident frequency correlation. In Figure 5(b), the ripples in the high-frequency region can be attributed to the S_21_ of the reference measurement link which drops in the high-frequency region. For each GPD, the measured frequency response indicates that the 3 dB bandwidth is much larger than 67 GHz and beyond the setup measurement range. The self-driven GPDs with large bandwidth features low power consumption and high-speed data reception. More details of the high frequency response measurements are given in Supplementary Note 5.

**Figure 6:**
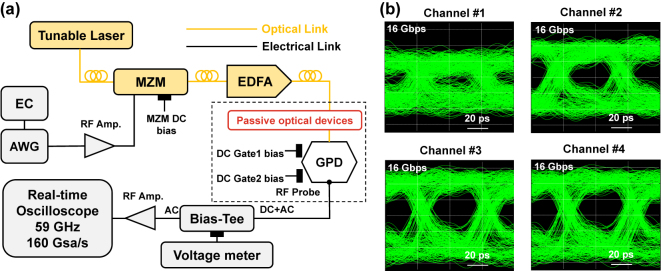
Demonstration of WDM optical data transmission by our optical receiver. (a) Measurement setup. EC: embedded controller, AWG: arbitrary waveform generator, MZM: Mach–Zehnder modulator. (b) Eye diagrams (0.01 mV div^−1^) of the 16 Gbps NRZ OOK data transmission by using four channels #1–#4, operating at zero-current bias. The modulated optical power input to the GPD is *P*_in_ = 2 mW.

Figure 6(a) depicts the measurement system of the high-speed optical data transmission, which includes an arbitrary waveform generator (AWG, M8195A), a commercial 40 GHz optical modulator, our WDM optical receiver, and a real-time oscilloscope (DSOZ594A). For each channel, the non-return-to-zero (NRZ) on-off-keying (OOK) signal generated by the AWG was amplified and injected to the optical modulator. The continuous wave light from tunable laser was modulated, amplified, and input to our receiver chip. The output signal of the GPD was amplified and then measured by the real-time oscilloscope. The time-domain signal is directly used to plot the eye diagram with no system optimization algorithm used. All the eye diagrams of the four channels are shown in Figure 6(b) with data rate of 16 Gbps. Under zero-current bias, each GPD operated with optimized gate voltages applied to maximize the responsivity. The modulated optical power into each GPD is *P*_in_ = ∼2 mW. The carrier wavelengths for four channels are respectively 1,550.25 nm, 1,548.7 nm, 1,546.4 nm, and 1,544.68 nm. Previously, we have demonstrated the PTE GPD operating in both 1.55 μm and 2 μm bands with similar performance based on this thin-Si waveguide platform [23]. In Supplementary Note 6, the operation of this GPD (including the mode converter) at 1.675 μm (U-band) is theoretically analysed, showing similar performance in contrast to that in C-band.

The static and high frequency performance of the four GPDs show good consistency, which can be attributed to the single 2DM stack strategy using a compact active region array. The 3 dB-bandwidths >67 GHz is comparable to the state-of-the-art works [24]. The MRRs of 0.5 nm bandwidth can support >30 Gbps NRZ data transmission theoretically. Therefore, we argue that the data transmission speed is limited by the responsivities of the GPDs.

In Supplementary Note 7, a performance comparison between the silicon waveguide integrated zero-biased PTE GPDs is given. The bandwidths of our GPDs reach the highest level among the counterparts. When comparing the responsivity on the mostly used 50 Ω load, the responsivities of our PTE GPDs are lower than two state-of-the-art works [22], [24] due to limited light absorption length, and outperform other recently reported works (see Supplementary Note 7). The ridge waveguide may cause strain in graphene, which degrades the responsivity of the PTE photoresponse [36]. In the future, more broadband WDM filters can be used, and the flat-top waveguide platform can be used to avoid the strain and non-ideal conformal contact between 2DM stack and the waveguide platform. As mechanical exfoliation technology continues to advance (e.g., large-area transfer method [37]), the GPD array with larger scale can be realized with equal and longer absorption lengths which mean better device uniformity and enhanced light absorption. Meanwhile, the top gates [22] can be used to avoid the imperfect Si electrodes. In this way, the device uniformity can be improved further, proving that our work holds significant potential for practical applications and represents a feasible technological approach.

## 4 Conclusions

In conclusion, we demonstrated a 4-channel WDM graphene optical receiver based on the MRR array and PTE GPDs operating at zero current. The MRRs realized a homogeneous spectral transmission at the bandwidth of 100 nm with ∼1.5-nm channel spacing. The active region array of the GPDs is specially designed within a compact footprint of ∼0.006 mm^2^, enabling the cover of a single high-quality mechanically exfoliated hBN/SLG/hBN stack. In this way, the GPDs show good consistency of the experimental performance. Specifically, the four GPDs exhibit similar responsivities of ∼1.1 V W^−1^ with flat frequency responses up to 67 GHz which is set-up limited. Using our graphene optical receiver, the transmission of 4 × 16 Gbps NRZ optical signal is demonstrated with carrier wavelengths of 1,550.25 nm, 1,548.7 nm, 1,546.4 nm, and 1,544.68 nm. This work can be easily extended to longer wavelengths, e.g. U-band, 2-μm-band or even beyond. To the best of our knowledge, this work represents the inaugural application of mechanically exfoliated high-quality graphene in the scale integration of GPDs, being a step forward in the application of graphene photonic devices in optical communications and interconnects. Apparently, our design is also compatible with CVD-grown graphene, whose compact active region array can result in an improved consistency of GPDs in contrast to the conventional designs. In the future, the waveguide platform with a flat top-interface can be employed to eliminate the impact of graphene strain on the responsivities of PTE GPDs, thereby enabling higher-speed data reception.

## 5 Methods

### 5.1 Transceiver fabrication

The thin silicon platform with 100-nm-thick Si layer was obtained from a standard SOI wafer with a 220-nm-thick top Si layer by using thermal oxidation and buffered oxide etch (BOE). The passive ridge silicon waveguides and circuits were fabricated by the processes of EBL and ICP dry-etching. For each GPD, the silicon slot ridge waveguide has a ridge width of 1.5 μm, a slot width of ∼120 nm, an etching depth of 100 nm. Next, the 90-nm-thick aluminum (30 nm)/Aurum (60 nm) gate-electrodes were fabricated by EBL, EBE and lift-off in acetone, forming ohmic contacts with two parts of the silicon slot waveguide separately. Then, a 10-nm-thick Al_2_O_3_ layer was deposited on the slot ridge waveguide by the atomic-layer deposition (ALD) process. The hBN/SLG/hBN stack was transferred onto the chip. Then, the Cr (5 nm)/Au (150 nm) electrodes were fabricated by EBL, EBE and lift-off processes, forming the edge contact between graphene and the source/drain electrodes. Finally, the hBN/SLG/hBN stack is patterned by the EBL and RIE etching processes to isolate the adjacent GPDs electrically.

### 5.2 Transfer process of graphene

The hBN/SLG/hBN stack was fabricated by van der Waals assembly technique. The graphene and hBN flakes were exfoliated from highly oriented pyrolytic graphite and hBN crystals using Stoch tape and heat release tape respectively. The graphite and hBN crystals were purchased from HQ Graphene Company. The hBN/SLG/hBN stack was picked up by a stamp composed of a polycarbonate (PC) layer supported by a polydimethylsiloxane (PDMS) block. For more details on the van der Waals assembly technique, please refer to ref. [38]. The assembled stack was released onto the active region array after oxygen plasma treatment.

## Supporting Information

Design of the micro-ring resonators, the multimode interferometer, and the strip-to-slot-waveguide mode converter. Details on graphene characterization. Details on the fabrication of the graphene optical receiver. Static performances of GPDs #2, #3, #4. Experimental setup for the high frequency response measurements. Theoretical analysis of the GPD operating in U-band. Performance comparison of the silicon waveguide integrated zero-biased graphene photodetectors.

## Supplementary Material

Supplementary Material Details

## References

[1] Cisco (2020). Cisco annual internet report (2018–2023) white paper. ..

[2] Shekhar S. (2024). Roadmapping the next generation of silicon photonics. *Nat. Commun.*.

[3] Atabaki A. H. (2018). Integrating photonics with silicon nanoelectronics for the next generation of systems on a chip. *Nature*.

[4] Michel J., Liu J., Kimerling L. C. (2010). High-performance Ge-on-Si photodetectors. *Nat. Photonics*.

[5] Hoshida T. (2022). Ultrawideband systems and networks: beyond C + L-band. *Proc. IEEE*.

[6] Taengnoi N., Bottrill K. R. H., Hong Y., Hanzo L., Petropoulos P. (2023). Ultra-long-span U-band transmission enabled by incoherently pumped Raman amplification. *J. Lightwave Technol.*.

[7] Baumgartner Y. (2021). High-speed CMOS-compatible III–V on Si membrane photodetectors. *Opt. Express*.

[8] Wen P. (2022). Waveguide coupled III–V photodiodes monolithically integrated on Si. *Nat. Commun.*.

[9] Massicotte M., Soavi G., Principi A., Tielrooij K.-J. (2021). Hot carriers in graphene – fundamentals and applications. *Nanoscale*.

[10] Bolotin K. I. (2008). Ultrahigh electron mobility in suspended graphene. *Solid State Commun.*.

[11] Liu C. (2021). Silicon/2D-material photodetectors: from near-infrared to mid-infrared. *Light Sci. Appl.*.

[12] Neumaier D., Pindl S., Lemme M. C. (2019). Integrating graphene into semiconductor fabrication lines. *Nat. Mater.*.

[13] Ding Y. (2020). Ultra-compact integrated graphene plasmonic photodetector with bandwidth above 110 GHz. *Nanophotonics*.

[14] Guo J. (2020). High-performance silicon−graphene hybrid plasmonic waveguide photodetectors beyond 1.55 μm. *Light Sci. Appl.*.

[15] Ma P. (2019). Plasmonically enhanced graphene photodetector featuring 100 Gbit/s data reception, high responsivity, and compact size. *ACS Photonics*.

[16] Wang Y. (2021). Ultrahigh-speed graphene-based optical coherent receiver. *Nat. Commun.*.

[17] Guo J., Liu C., Yu L., Xiang H., Xiang Y., Dai D. (2023). High-speed graphene–silicon–graphene waveguide PDs with high photo-to-dark-current ratio and large linear dynamic range. *Laser Photonics Rev.*.

[18] Shiue R.-J. (2015). High-responsivity graphene–boron nitride photodetector and autocorrelator in a silicon photonic integrated circuit. *Nano Lett.*.

[19] Schuler S. (2016). Controlled generation of a p–n junction in a waveguide integrated graphene photodetector. *Nano Lett.*.

[20] Marconi S. (2019). Waveguide integrated CVD graphene photo-thermo-electric detector with >40GHz bandwidth. *Conference on Lasers and Electro-Optics*.

[21] Mišeikis V. (2020). Ultrafast, zero-bias, graphene photodetectors with polymeric gate dielectric on passive photonic waveguides. *ACS Nano*.

[22] Schuler S. (2021). High-responsivity graphene photodetectors integrated on silicon microring resonators. *Nat. Commun.*.

[23] Yu L. (2023). High-bandwidth zero-biased waveguide-integrated p-n homojunction graphene photodetectors on silicon for a wavelength band of 1.55 μm and beyond. *ACS Photonics*.

[24] Marconi S. (2021). Photo thermal effect graphene detector featuring 105 Gbit s^−1^ NRZ and 120 Gbit s^−1^ PAM4 direct detection. *Nat. Commun.*.

[25] Akinwande D. (2019). Graphene and two-dimensional materials for silicon technology. *Nature*.

[26] Wang L. (2013). One-dimensional electrical contact to a two-dimensional material. *Science*.

[27] Ma L., Ren W., Cheng H. (2019). Transfer methods of graphene from metal substrates: a review. *Small Methods*.

[28] Giambra M. A. (2021). Wafer-scale integration of graphene-based photonic devices. *ACS Nano*.

[29] Schall D., Porschatis C., Otto M., Neumaier D. (2017). Graphene photodetectors with a bandwidth >76 GHz fabricated in a 6″ wafer process line. *J. Phys. D Appl. Phys.*.

[30] Alessandri C. (2020). 5 × 25 Gbit/s WDM transmitters based on passivated graphene–silicon electro-absorption modulators. *Appl. Opt.*.

[31] Yu L., Guo J., Xiang H., Liu C., Zhao Y., Dai D. (2024). High-performance 2 × 2 bent directional couplers designed with an efficient semi-inverse design method. *J. Lightwave Technol.*.

[32] Shahrokhi M., Mortazavi B., Berdiyorov G. R. (2017). New two-dimensional boron nitride allotropes with attractive electronic and optical properties. *Solid State Commun.*.

[33] Song J. C. W., Rudner M. S., Marcus C. M., Levitov L. S. (2011). Hot carrier transport and photocurrent response in graphene. *Nano Lett.*.

[34] Gabor N. M. (2011). Hot carrier–assisted intrinsic photoresponse in graphene. *Science*.

[35] Tielrooij K. (2015). Generation of photovoltage in graphene on a femtosecond timescale through efficient carrier heating. *Nat. Nanotechnol.*.

[36] Zhao Y. (2022). Large-area transfer of two-dimensional materials free of cracks, contamination and wrinkles via controllable conformal contact. *Nat. Commun.*.

[37] Moon J. Y. (2020). Layer-engineered large-area exfoliation of graphene. *Sci. Adv.*.

[38] Purdie D. G., Pugno N. M., Taniguchi T., Watanabe K., Ferrari A. C., Lombardo A. (2018). Cleaning interfaces in layered materials heterostructures. *Nat. Commun.*.

