# Supplementary material for: Four-channel graphene optical receiver
Source: Nanophotonics. 2024 Jul 31;13(21):4019–28. doi: 10.1515/nanoph-2024-0274 (PMC11501057; doi:10.1515/nanoph-2024-0274)
Supplement: Supplementary file 1 — Supplementary Material Details [file j_nanoph-2024-0274_suppl_001.docx]

**Supplementary Information**

**Four-Channel Graphene Optical Receiver**

**Supplementary Note 1. Design of the micro-ring resonators, the multimode interferometer, and the** **strip-to-slot-waveguide mode converter.**

The micro-ring resonator (MRR) array forms a 4-channel wavelength demultiplexing filter with a waveguide height of 100 nm. Fig. S1(a) depicts the structure of one MRR, including a pair of Bezier-shaped couplers [1]. The transmission spectra of each MRR are obtained by using the transfer matrix method based on the phase shift of the ring and the transmission spectra of the couplers. The couplers are designed by the semi-inverse design (SID) method introduced in our previous works [1,2] using the finite-difference time-domain (FDTD) method. The two couplers of the add-drop MRR denoted by the orange waveguides in Fig. S1(a) are placed centro-symmetrically. The design process of one coupler is given below.


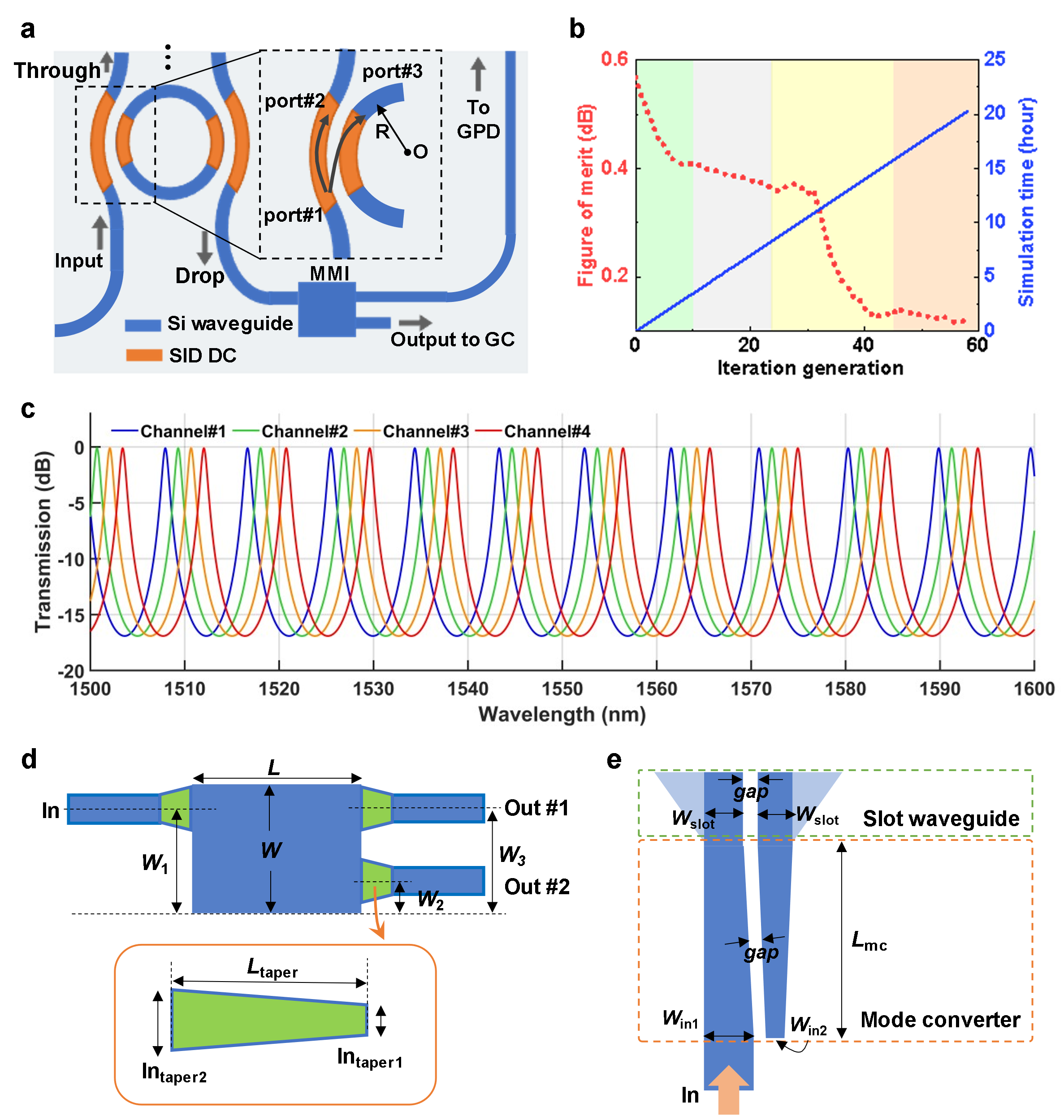


**Figure S1:** Design of the passive photonic devices. (a) The MRR structure and the definition of the couplers. (b) Figure of merit as a function of the iteration generation for the couplers with design wavelength range of 1.5-1.6 μm. (c) The simulated drop-port transmission spectra of the designed four-channel MRR array. (d) Structure of the 90%:10% MMI. (e) Structure of the strip-to-slot-waveguide mode converter.

The optimization figure of merit (*FOM*) for a specifical wavelength *λ_i_* is defined by [1]:

${FOM}_{\mathrm{in}}(\lambda_{i})=-10\log_{10}\{1-\sqrt{\left[ CR-\left| S_{31}\left( \lambda_{i} \right) \right|^{2} \right]^{2}+\left[ 1-CR-\left| S_{21}\left( \lambda_{i} \right) \right|^{2} \right]^{2}}\}$, (S1)

where *S*_xy_ is the coupling coefficient from mode #*y* to mode #*x*, and *CR* is the target power coupling ratio. Here modes #1-#3 respectively refer to the TE_0_ modes of ports #1-#3. To design a broadband directional coupler for the MRR, multiple operating wavelengths were considered:

$FOM=\frac{1}{n}\sum_{i=1}^{n} {FOM}_{\mathrm{in}}(\lambda_{i})$. (S2)

We choose five wavelengths (*n* = 5) as {1.5, 1.525, 1.55, 1.575, 1.6} μm and *CR* = 0.25 to make the MRR work well in a broadband. As Fig. S1(b) shows, the *FOM* varies from 0.57 to 0.11 within 59 generations in the three stages of optimization, taking ~20 hours totally. By fine tuning the ring radius, the four MRRs were designed with the same free spectral range (FSR) and different resonance wavelengths. Fig. S1(c) shows the simulated drop-port transmission spectra of the four-channel MRRs, each of which shows a 1-dB bandwidth of ~0.5 nm. As Fig. S1(a) shows, the optical signal output from drop port was guided to the multimode interferometer (MMI) power splitter with 90%:10% splitting ratio. Fig. S1(d) depicts the structure of the MMI with the parameter definitions. The optimized parameters are listed in Table S1. The structure of the strip-to-slot-waveguide mode converter is given in Fig. S1(e) with the optimized parameters given in Table S2.

**Table S1:** Design parameters of the MMI.

| W (μm) | L (μm) | W_1_ (μm) | W_2_ (μm) | W_3_ (μm) | In_taper1_ (μm) | In_taper2_ (μm) | L_tapper_ (μm) |
| --- | --- | --- | --- | --- | --- | --- | --- |
| 2.8 | 30.8 | 2.1 | 0.75 | 2.2 | 1 | 1.35 | 2 |

**Table S2:** Design parameters of the strip-to-slot-waveguide mode converter.

| W_in1_ (μm) | W_in2_ (μm) | W_slot_ (μm) | gap (μm) | L_mc_ (μm) |
| --- | --- | --- | --- | --- |
| 0.7 | 0.3 | 0.6 | 0.15 | 24.5 |

**Supplementary Note 2. Details on graphene characterization.**

As shown in Fig. S2, we performed a Raman Spectrometer (LabRAM HR Evolution, HORIBA, Japan, wavelength 532 nm, power<1 mW) and an atomic force microscopy (AFM, dimension ICON, Bruker, Germany) to monitor the SLG quality at the position of the final device. A typical Raman spectrum before further processing of the stack is shown in Fig. S2(a). The position of the combined hBN E_2g_ peaks [3] from top and bottom flakes is Pos(E_2g_) ~1365 cm^−1^ with a full-width half maximum FWHM(E_2g_) of ~9 cm^−1^, as expected considering the top flake is bulk and that the planar domain size is limited by the flake size [4]. Pos(2D) ~2683 cm^−1^, FWHM(2D) ~20.1 cm^−1^, Pos(G) ~ 1582 cm^−1^, and FWHM(G) ~ 12 cm^−1^ confirm the presence of SLG. The AFM images of the slot waveguide and the active region with 2DM stack are shown in Fig. S2(b) and Fig. S2(c), respectively. It can be seen that the 2DM stack atop the ridge slot waveguide is not a perfect conformal fit. Nevertheless, the maximum undulating height in the active region (~132 nm, in Fig. S2(c)) and the passive waveguide area (~135 nm, in Fig. S2(b)) is basically the same, indicating that the stack fits on the waveguide well.

**Figure S2:** Characterization of the 2DM stack. (a) Raman spectrum measured at the position of the final device. (b) AFM image of the slot waveguide. (c) AFM image of the hBN/SLG/hBN atop the waveguide.

A reference field effect transistor (FET) was fabricated along with the graphene PDs, consisting of hBN/SLG/hBN channel with *L*_c_ = 4.5 μm and *L*_g_ = 30 μm. This FET shares the same 10-nm-thick Al_2_O_3_ and 15-nm-thick bottom hBN insulator layer with the graphene PDs. The total resistance between the drain and source electrodes (*R*_tot_) was measured as gate voltage (*V*_G_) varies, as shown in Fig. S1. As introduced in our previous work, the total resistance of the graphene can be expressed as:

$R_{tot}=R_{c}+R_{g}=R_{c}+\frac{L_{c}}{L_{g}}\sigma^{-1}$, (S3)

where *R*_c_ is the total resistance of the metal-graphene contacts, *R*_g_ is the resistance of gate-tunable graphene, *σ* is the electrical conductivity of graphene. The graphene electrical conductivity *σ* can again be expressed by

$\sigma=\sqrt{\sigma_{min}^{2}+[\mu C_{G}(V_{G}-V_{Dirac})]}$, (S4)

where *σ*_min_ is graphene minimum conductivity, *μ* is the carrier mobility of graphene, *V*_Dirac_ is the gate voltage corresponding to the charge neutral point of graphene, and *C*_G_ is the gate dielectric capacitance given by [4, 5] $C_{G}={{(C}_{{Al}_{2}O_{3}}^{-1}+C_{hBN}^{-1})}^{-1}=8.83\times{10}^{-4} F m^{-2}$. By fitting the measured *R*_tot_-*V*_G_ curve with Eqs. S3 and S4, we have *R*_c_ = ~65 Ω, *σ*_min_ = ~2.6 mS, *μ* = ~5.06×10^4^ cm^2^·V^-1^·s^-1^ and *V*_Dirac_ = −1 V. The fitting curve is shown by the red solid line in Fig. S3.

**Figure S3:** Electrical characterization of a reference field effect transistor (FET). Blue dots: measured data, solid line: fitting result.

**Supplementary Note 3.** **Details on the fabrication of the graphene optical receiver.**

Figure S4 illustrates the fabrication process of our graphene optical receiver. Firstly, the SOI with 100 nm silicon top layer was obtained from a standard SOI wafer with a 220-nm-thick top silicon layer by using thermal oxidation and buffered oxide etch (BOE) processes. The full-etched (100 nm) and shallow-etched (50 nm) Si photonic waveguides are defined by two round of electron beam lithography (EBL) and inductively-coupled plasma dry-etching (ICP) processes, as shown in Fig. S4(a). The fabrication of the GPD active regions is shown in Figs. S4(b)-(f). The 90-nm-thick Al (30 nm)/Au (60 nm) gate-electrodes were fabricated by processes of EBL, electron-beam evaporation (EBE), and lift-off in acetone, forming ohmic contacts with two parts of the silicon slot waveguide separately. Then, a 10-nm-thick Al_2_O_3_ layer was deposited on the slot ridge waveguide by the atomic-layer deposition (ALD) process (Fig. S4(c)). The hBN/SLG/hBN stack was transferred onto the active region area (Fig. S4(d)). The 2DM stack was patterned by the EBL and RIE processes, followed by the fabrication of Cr (5 nm)/Au (150 nm) source and drain electrodes, in this way, the metal-SLG edge contacts were formed (Fig. S4(e)). Finally, the part of the 2DM stack between the adjacent GPDs was removed by oxygen plasma etching to ensure that each GPD works independently.

**Figure S4:** Fabrication process of the graphene optical receiver. (a) Fabrication of the Si full-etched and shallow-etched waveguides. (b) Al/Au gate electrodes fabrication by EBL, EBE, and lift-off processes. (c) Deposition of the Al_2_O_3_ dielectric layer. (d) hBN/SLG/hBN stack transfer. (e) Cr/Au-SLG edge conduct forming. (f) The oxygen plasma etching of the hBN/SLG/hBN stack for electrical isolation of the adjacent GPDs.

**Supplementary Note 4. Static performances of GPDs #2, #3, #4.**

As shown in Fig. S4(a)-(c), the resistance of the graphene channel under different gate voltages was measured under near-zero bias voltage of 1 mV, showing that the charge neutrality point voltage *V*_CNP_ is about −1 V. Figure S4d-f give the measured *V*_PTE_ under zero bias at the wavelength of 1548.7 nm, 1546.4 nm, 1544.68 nm with *P*_in_ = ~0.1 mW as a function of the gate voltages (*V*_G1_, *V*_G2_) for GPDs #2, #3, #4.

**Figure S5:** Experimental static performances of GPDs #2, #3, #4. (a)-(c) Measured resistance map of the fabricated PTE GPD #2 a, #3 b, #4 (c) as a function of the gate voltages V_G1_ and V_G2_; (d)-(f) Measured photovoltage maps at 1548.7 nm, 1546.4 nm, 1544.68 nm with *P*_in_ = ~0.1 mW under zero-current bias for GPD #2 (d), #3 (e), #4 (f).

**Supplementary Note 5. Experimental setup for the high frequency response measurements.**

Figure S6 shows the experimental setup for frequency response measurements. Firstly, we calibrated the bias-Tee and cables in the test system. The optical signals generated by a tunable laser (TL) at λ_CH#n_ (λ_CH#1,2,3,4_ = 1550.25 nm, 1548.7 nm, 1546.4 nm and 1544.68 nm) was modulated by an optical modulator integrated in a light-wave component analyzer (LCA, KEYSIGHT N4373E). The modulated optical signal was then amplified by an EDFA and coupled to the grating coupler. In each channel, the light passed through a power splitter (90%/10%) with a multimode interferometer (MMI) structure. The 10%-power optical signal was given to the commercial photodetector (integrated in the LCA), forming a reference calibration path. In this way, the frequency responses of the MRR, bias-Tee and optical modulator can be calibrated. The AC photocurrent signal generated by the GPD was then extracted by a bias-Tee (SHF BT 65-B) and amplified by an RF amplifier (SHF 807C). Then, the LCA received the amplified AC signal and gives the *S*_21_ response. Finally, the high frequency responses of the RF probe and RF amplifier were calibrated to obtain the *S*_21_ response of the GPD.

**Figure S6:** Detailed experimental set up for the high frequency response measurement. TL: Tunable laser; EDFA: Erbium-doped fiber amplifier; LCA: Light-wave component analyzer. PS: Power splitter. OM: optical modulator.

**Supplementary Note 6. Theoretical analysis of the GPD operating in U-band.**

Fig. S7 shows the simulation result of the GPD operating at 1.675 μm. Fig. S7(a) shows the mode-field distribution in the slot cross-section, with an effective refractive index of 1.8621－0.0045*i*, corresponding to a mode absorption coefficient of about ~0.23 dB μm^−1^. The transmission optical filed of the mode converter is depicted in Fig. S7(b). Given that the mode converter was optimized for C-band, a larger loss of ~1.6 dB is obtained at 1.675 μm, which can be reduced further with longer mode conversion structure. As demonstrated in our previous work [6], the thin-Si platform supports the development of PTE GPDs operation at both 1.55 μm and 2 μm. The photoresponse at 1.675 μm was also simulated with the result shown in Fig. S7(c). The responsivity of ~1.85 V W^-1^ is almost the same with that of at 1.55 μm. This analysis confirms the wavelength scalability of the thin-Si platform.

**Figure S7:** Theoretical results of the GPD operating in U-band (at 1.675 μm). (a) Transversal electric field distributions ($|E_{T}|=\sqrt{{|E_{x}|}^{2}+{|E_{y}|}^{2}}$) of the fundamental quasi-TE mode for the optimized slot ridge waveguide and graphene sheet, the normalized mode power is 1 mW. (b) Normalized simulated light propagation in the mode converter when operating at 1.675 μm. (c) Six-fold photovoltage responsivity map of the designed GPD.

**Supplementary Note 7. Performance comparison of the silicon waveguide integrated zero-biased graphene photodetectors.**

Table S3 gives a comparison for the zero-biased waveguide-integrated GPDs based on the PTE effect [6,7,8,9,10,11,-12], most of which operated at ~1.55 μm [6,7,8,9,10,-11]. These zero-biased PTE GPDs are self-driven with zero power consumption theoretically. While the open-circuit responsivities (*R*_oc_) were usually measured and reported in the articles, the 50-Ω-load responsivity is a more important indicator, which represents the conversion efficiency between the actual electrical signal strength read by the driving circuit and the input optical power, given by *R*_50Ω_= *R*_oc_·50/(*R*_tot_+50), where *R*_tot_ denotes the total resistance of the GPD. As Table S3 shows, two GPDs based on mechanical exfoliated graphene can result in *R*_50Ω_ over 1 V/W [7,8], benefiting from the light absorption enhancement structures. Specifically, a state-of-the-art PTE GPD with *R*_oc_ of 90 V/W and *R*_50Ω_ of 5.2 V/W [8] can be used to construct the optical receiver (with proper TIA) with sensitivity on par with the SiGe counterparts. However, the bandwidths are limited to 10-18 GHz in refs. [7,8], and no data transmission was demonstrated. In contrast, our GPDs based on the thin-Si platform with no optical resonant structure still exhibit acceptable *R*_50Ω_ of ~0.5 V/W with much better high frequency performance. Besides, our GPDs exhibit larger *R*_50Ω_ than most of the counterparts using CVD-grown graphene [6,9,10,12], which were reported with no demonstration of data transmission. Currently, the state-of-the-art PTE GPD using flat waveguide interface and top graphene gate electrodes showcased large *R*_50Ω_ of 1.25 V/W and bandwidth over 67 GHz, demonstrating the 105 Gbps NRZ single transmission [11]. As Table S1 shows, its graphene absorption length of 50 μm is much longer than our work (~27-30 μm).

In summary, the responsivities of our PTE GPDs are higher than most recently reported works, and lower than two state-of-the-art works due to limited light absorption length and lack of optical resonance structure. The bandwidths of our PTE GPDs reach the highest level among the counterparts. Although the data rate is limited by the device responsivity and the loss in the photonic integrated circuit, the data transmission demonstration is quite valuable, given that it is not provided frequently.

Table S3 Comparison of the recently reported zero-biased waveguide-integrated PTE GPDs.

| Ref. | Year | λ (μm) | Graphene type | Absorption  Length (μm) ^a)^ | Channel Length (μm) | Resistance (Ω) | *R*_oc_^b)^ (V/W) | *R*_50Ωload_ ^c)^ (V/W) | Bandwidth (GHz) | Data rate (Gbps) |
| --- | --- | --- | --- | --- | --- | --- | --- | --- | --- | --- |
| [7] | 2018 | 1.55 | Flake^d)^ | 40 | 3 | ~180 | 4.7 | 1.02 | 18 | - |
| [8] | 2021 | 1.55 | Flake | 6 | 2.5 | ~800 | 90 | 5.2 | 10 | - |
| [9] | 2019 | 1.55 | CVD | 20 | 3 | ~6000 | 12.2 | 0.1 | 42 | - |
| [10] | 2020 | 1.55 | CVD | 80 | 3.5 | ~750 | 6 | 0.375 | >67 | - |
| [11] | 2021 | 1.55 | CVD | 50 | 1.5 | ~90 | 3.5 | 1.25 | 65 | 105/120 |
| [12] | 2022 | 5.2 | CVD | - | 5.4 | ~170 | 1.5 | 0.34 | >1 MHz | - |
| [6] | 2023 | 1.55 | CVD | 60 | 10 | ~1500 | 2.81 | 0.09 | >40 | - |
|  |  | 2 | CVD | 60 | 10 | ~1500 | 2.78/4.19 | 0.09/0.13 | >22 | - |
| This work | 2024 | C band | Flake | ~27-30 | 4.5 | ~60 | ~1-1.3 | 0.45-0.59 | >67 | 16 |

Notes:

a) The light absorption enhancement structures based on slow light waveguide and MRR were used in ref. [6] and ref. [8], respectively.

b) *R*_oc_ denotes the responsivity in open circuit mode.

c) *R*_50Ωload_ denotes the responsivity when a 50 Ω load is used, which corresponds to the vast majority of real operation condition.

d) The graphene flake is based on mechanical exfoliation method.

# Supplementary References

1. L. Yu *et al.*, “High-Performance 2 × 2 Bent Directional Couplers Designed With an Efficient Semi-Inverse Design Method,” *J. Lightwave Technol.*, vol. 42, pp. 740–747, 2024.
2. J. Guo *et al.*, “Realization of Advanced Passive Silicon Photonic Devices with Subwavelength Grating Structures Developed by Efficient Inverse Design,” *Adv. Photon. Nexus*, vol. 2, p. 026005, 2023.
3. S. Reich *et al.*, “Resonant Raman Scattering in Cubic and Hexagonal Boron Nitride,” *Phys. Rev. B*, vol. 71, p. 205201, 2005.
4. D. G. Purdie *et al.*, “Cleaning Interfaces in Layered Materials Heterostructures,” *Nat. Commun.*, vol. 9, p. 5387, 2018..
5. J. Guo *et al.*, “High-performance silicon−graphene hybrid plasmonic waveguide photodetectors beyond 1.55 μm,” *Light Sci. Appl.*, vol. 9, p. 29, 2020.
6. L. Yu *et al.*, “High-Bandwidth Zero-Biased Waveguide-Integrated p-n Homojunction Graphene Photodetectors on Silicon for a Wavelength Band of 1.55 μm and Beyond,” *ACS Photonics*, vol. 10, pp. 3621–3628, 2023.
7. S. Schuler *et al*. “Graphene Photodetector Integrated on a Photonic Crystal Defect Waveguide,” *ACS Photonics*, vol. 5, pp. 4758–4763, 2018.
8. S. Schuler *et al.*, “High-responsivity graphene photodetectors integrated on silicon microring resonators,” *Nat. Commun.*, vol. 12, p. 3733, 2021.
9. J. Muench *et al*., “Waveguide-Integrated, Plasmonic Enhanced Graphene Photodetectors,” *Nano Lett.*, vol. 19, pp. 7632–764, 2019.
10. V. Mišeikis *et al.*, “Ultrafast, Zero-Bias, Graphene Photodetectors with Polymeric Gate Dielectric on Passive Photonic Waveguides,” *ACS Nano*, vol. 14, pp. 11190–11204, 2020.
11. S. Marconi *et al.*, “Photo thermal effect graphene detector featuring 105 Gbit s^−1^ NRZ and 120 Gbit s^−1^ PAM4 direct detection,” *Nat. Commun.*, vol. 12, p. 806, 2021.
12. J. Goldstein *et al*., “Waveguide-integrated mid-infrared photodetection using graphene on a scalable chalcogenide glass platform,” *Nat. Commun.*, vol. 13, p. 3915, 2022.
